# Krempfielins J–M, New Eunicellin-Based Diterpenoids from the Soft Coral *Cladiella krempfi*

**DOI:** 10.3390/md11082741

**Published:** 2013-08-02

**Authors:** Yan-Ning Lee, Chi-Jen Tai, Tsong-Long Hwang, Jyh-Horng Sheu

**Affiliations:** 1 Department of Marine Biotechnology and Resources, National Sun Yat-sen University, Kaohsiung 80424, Taiwan; E-Mails: jennyyanningl@yahoo.com.tw (Y.-N.L.); jean801023@hotmail.com (C.-J.T.); 2 Graduate Institute of Natural Products, Chang Gung University, Taoyuan 33302, Taiwan; E-Mail: htl@mail.cgu.edu.tw; 3 Division of Marine Biotechnology, Asia-Pacific Ocean Research Center, National Sun Yat-sen University, Kaohsiung 80424, Taiwan; 4 Department of Medical Research, China Medical University Hospital, China Medical University, Taichung 40402, Taiwan; 5 Graduate Institute of Natural Products, Kaohsiung Medical University, Kaohsiung 80708, Taiwan

**Keywords:** *Cladiella krempfi*, eunicellin-based diterpenoid, elastase, anti-inflammatory agent

## Abstract

New four eunicellin-based diterpenoids, krempfielins J–M (**1**–**4**) were isolated from the organic extract of a Taiwanese soft coral *Cladiella krempfi*. The structures of the new metabolites were elucidated on the basis of extensive spectroscopic analysis. The structure of compound **2** is rare due to the presence of the highly oxygenated pattern. Anti-inflammatory activity of **1**–**6** to inhibit the superoxide anion generation and elastase release in FMLP/CB-induced human neutrophils was also evaluated, and **2** and **4** were shown to possess the ability to inhibit the elastase release.

## 1. Introduction

**Chart 1:**
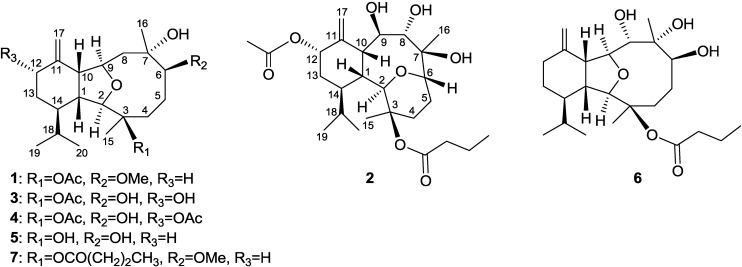
Structures of metabolites **1**–**6**.

Many recent studies about the discovery of versatile structures and bioactivities of eunicellin-type compounds isolated from soft corals have been reported [1,2,3,4,5,6,7,8,9,10,11,12,13,14,15]. The soft coral *Cladiella krempfi* has been found to generate several types of metabolites including eunicellin-type diterpenoids [16,17] and pregnane-type steroids [18,19,20]. Our previous study on the bioactive secondary metabolites of a Taiwanese soft coral *Cladiella krempfi* also resulted in the isolation of a series of new eunicellin-based diterpenoids, krempfielins A–I [21,22]. In this paper, we further report the discovery of four new eunicellin-based diterpenoids, krempfielins J–M (**1**–**4**), along with two known diterpenoids sclerophytin F (**5**) [23] and litophynol B (**6**) [24] (Chart 1). The ability of these compounds to inhibit the superoxide anion generation and elastase release in FMLP/CB-induced human neutrophils was also evaluated. The results showed that at 10 μM compounds **2** and **4** effectively inhibited the elastase release in FMLP/CB-induced human neutrophils.

## 2. Results and Discussion

Krempfielin J (**1**) showed the pseudomolecular ion peak [M + Na]^+^ at *m*/*z* 417.2616 in the HRESIMS and the molecular formula was determined as C_23_H_38_O_5_. NMR spectroscopic data of **1** (Table 1) showed the presence of one acetoxy group (δ_C_ 169.6 and 22.4; δ_H_ 2.12, s, 3H) and one methoxyl group (δ_C_ 57.0 and δ_H_ 3.34, 3H, s). The NMR data of **1** was found to be similar to the known compound, **7** [22,25] (Chart 1). Comparison of the NMR data of them revealed that the only difference between both compounds arises from the replacement of the *n*-butyryloxy group at C-3 in **7** by one acetoxy group in **1**. The stereochemistry of **1** was confirmed by comparison of the NMR data and NOE correlations of both **7** and **1**.

**Table 1:** ^13^C and ^1^H NMR data for compounds **1**–**4**.

	1 ^a^		2 ^b^		3 ^a^		4 ^a^	
	δ_C_	δ_H_	δ_C_	δ_H_	δ_C_	δ_H_	δ_C_	δ_H_
1	45.6, CH ^c^	2.20 dd	37.8, CH	3.24 m	44.2, CH	2.31 t (8.0)	44.2, CH	2.31 ddd
		(10.8, 8.0) ^d^						(8.0, 6.0, 2.0)
2	92.1, CH	3.59 br s	80.9, CH	3.58 m	90.4, CH	3.69 br s	90.2, CH	3.69 d (2.0)
3	86.6, C		83.8, C		86.6, C		86.6, C	
4	36.8, CH_2_	1.75 m	28.8, CH_2_	1.92 m	34.6, CH_2_	1.94 m	34.9, CH_2_	1.92 m
		2.61 dd		2.90 dd		2.48 m		2.52 dd
		(14.4, 9.2)		(14.8, 7.0)				(14.8, 8.8)
5	26.8, CH_2_	1.33 m	19.9, CH_2_	1.50 m	30.4, CH_2_	1.42 m	30.5, CH_2_	1.45 m
		1.59 m		1.71 m		1.71 m		1.68 m
6	90.6, CH	4.12 d (6.0)	70.1, CH	3.68 m	79.0, CH	4.57 d (8.4)	79.1, CH	4.58 d (7.2)
7	75.9, C		75.5, C		76.6, C		76.5, C	
8	45.0, CH_2_	1.82 m	79.0, CH	3.62 m	46.0, CH_2_	1.85 m	45.9, CH_2_	1.83 m
9	78.5, CH	4.18 dd	69.9, CH	4.39 t (8.0)	79.4, CH	4.53 m	78.8, CH	4.40 m
		(14.8, 7.2)						
10	53.9, CH	2.95 t (7.2)	50.0, CH	2.51 br s	51.0, CH	2.86 m	51.4, CH	2.93 dd
								(7.2, 6.0)
11	147.6, C		145.3, C		148.0, C		143.0, C	
12	31.5, CH_2_	2.04 m	71.6, CH	5.29 m	70.7, CH	4.37 s	72.5, CH	5.44 dd
		2.25 m						(5.6, 2.8)
13	24.6, CH_2_	1.00 m	31.9, CH_2_	1.58 m	31.2, CH_2_	1.46 m	29.0, CH_2_	1.42 m
		1.70 m		2.17 m		1.84 m		1.90 m
14	44.0, CH	1.26 m	40.1, CH	1.48 m	36.5, CH	1.82 m	37.2, CH	1.70 m
15	23.1, CH_3_	1.41 s	26.3, CH_3_	1.56 s	23.2, CH_3_	1.48 s	23.2, CH_3_	1.47 s
16	23.6, CH_3_	1.13 s	13.7, CH_3_	1.19 s	22.6, CH_3_	1.17 s	22.8, CH_3_	1.18 s
17	109.4, CH_2_	4.64 s	105.0, CH_2_^e^	5.04 s	111.8, CH_2_	4.87 s	114.9, CH_2_	4.97 s
		4.68 s		5.23 s		5.06 s		5.14 s
18	29.1, CH	1.72 m	28.3, CH	2.03 m	29.0, CH	1.80 m	28.9, CH	1.80 m
19	15.6, CH_3_	0.79 d (6.8)	21.5, CH_3_	1.06 d (6.5)	16.4, CH_3_	0.85 d (6.4)	16.2, CH_3_	0.83 d (6.8)
20	21.9, CH_3_	0.97 d (6.8)	21.5, CH_3_	1.09 d (6.5)	21.9, CH_3_	1.00 d (6.4)	21.8, CH_3_	0.96 d (6.8)
3-*n*-butyrate			172.6, C					
			37.5, CH_2_	2.22 m				
				2.30 m				
			18.6, CH_2_	1.64 m				
			13.8, CH_3_	0.95 t (7.5)				
3-OAc	169.6, C				169.5, C		169.4, C	
	22.4, CH_3_	2.12 s			22.4, CH_3_	2.07 s	22.4, CH_3_	2.07 s
6-OMe	57.0, CH_3_	3.34 s						
6-OAc								
8-OAc								
12-OAc			170.0, C				170.3, C	
			21.2, CH_3_	2.14 s			21.5, CH_3_	2.06 s

^a^ ^13^C and ^1^H spectra recorded at 100 and 400 MHz in CDCl_3_; ^b^ ^13^C and ^1^H spectra recorded at 125 and 500 MHz in CDCl_3_; ^c^ Deduced from DEPT; ^d^ *J* values (Hz) in parentheses; ^e^ Broad signal.

**Figure 1:**
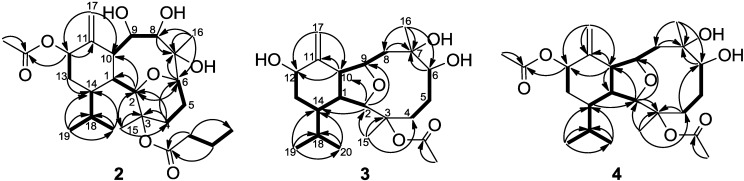
Selected ^1^H-^1^H COSY (▬) and HMBC (→) correlations of **2**–**4**.

**Figure 2:**
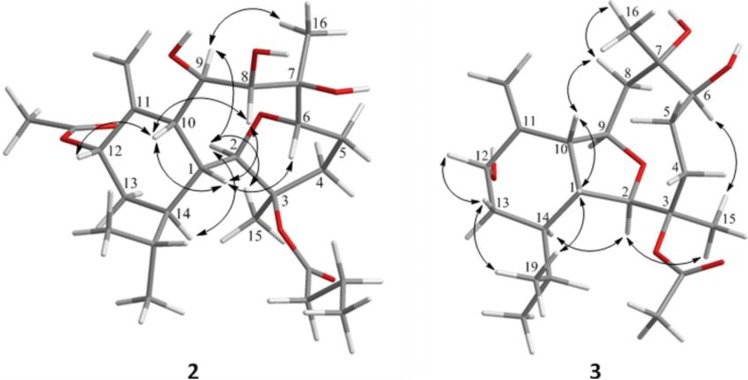
Key NOESY correlations for **2** and **3**.

The new metabolite krempfielin K (**2**) was found to have the molecular formula C_26_H_42_O_8_ and six degrees of unsaturation, as indicated from the HRESIMS. The IR absorptions at ν_max_ 3444 and 1732 cm^−1^ revealed the presence of hydroxy and ester carbonyl functionalities. The ^13^C carbons spectral data (Table 1) were assigned by the assistance of DEPT spectrum to six methyls (including one acetate methyl δ_C_ 21.2), five sp^3^ methylenes, one sp^2^ methylene, nine sp^3^ methines (including five oxymethines), two sp^3^ and three sp^2^ quaternary carbons (including two ester carbonyls). The NMR spectroscopic data of **2** (Table 1) showed the presence of one 1,1-disubstituted double bond (δ_C_ 105.0, CH_2_ and 145.3, C; δ_H_ 5.23, s and 5.04, s, each 1H). Two ester carbonyls (δ_C_ 172.6 and 170.0) were assigned from the ^13^C NMR spectrum and were HMBC correlated with the methylene (δ_H_ 2.30 m and 2.22 m, each 1H) of an *n*-butyrate and those of an acetate methyl (δ_H_ 2.14, s, 3H), respectively. Therefore, the remaining three degrees of unsaturation identified compound **2** as a tricyclic molecule. ^1^H-^1^H COSY and HMBC correlations (Figure 1) were further used to establish the molecular skeleton of **2**. The COSY experiment assigned five isolated consecutive proton spin systems. Above evidences and HMBC correlations from H-2 and H-6 (δ_H_ 3.58 and 3.68) to C-6 and C-2 (δ_C_ 70.1 and 80.9), respectively, implied the presence of an ether linkage between C-2 and C-6, and suggested that **2** is an 2,6-ether linked eunicellin-based diterpenoid [1,26]. Furthermore, the acetoxy group attaching at C-12 was confirmed from the HMBC correlations from H-12 (δ_H_ 5.29) and acetate methyl protons (δ_H_ 2.14) to the carbonyl carbon appearing at 170.0 (C). Thus, the remaining one *n*-butyryloxy group had to be positioned at C-3, an oxygen-bearing quaternary carbon resonating at δ 83.8 ppm. On the basis of above analysis, the planar structure of **2** was established. The relative structure of **2** was elucidated by the analysis of NOESY correlations, as shown in Figure 2. The observation of the NOE interactions between H-1 and H-6, H-8 and H-10; H-10 and both H-8 and H-12, revealed that they are all β-oriented. Also, the correlations between H-2 and H-9, H-14 and H_3_-15; H-9 and H_3_-16 suggested that of all of H-2, H-9, H-14, H_3_-15 and H_3_-16 are α-oriented. The relative configuration of **2** was thus established. The highly oxygenated pattern of **2** at C-2, C-3, C-6, C-7, C-8, C-9 and C-12 is rare in known eunicellins.

Krempfielin L (**3**) showed the molecular ion peak [M + Na]^+^ at *m*/*z* 419.2407 in the HRESIMS and established a molecular formula of C_22_H_36_O_6_, implying five degrees of unsaturation. The IR absorptions at ν_max_ 3419 and 1734 cm^−1^ revealed the presence of hydroxy and ester carbonyl functionalities. The ^13^C NMR spectrum of **3** showed signals of 22 carbons (Table 1), which were characterized by the DEPT spectrum as five methyls (including one acetate methyl), five methylenes (including one sp^2^ methylene), eight methines (including four oxygenated carbons), and four quaternary carbons (including one ester carbonyl and one sp^2^ quaternary carbon of an olefin). The ^1^H and ^13^C NMR spectral data of **3** (Table 1) also showed the presence of one acetoxy group (δ_H_ 2.07, s, 3H; δ_C_ 22.4, CH_3_ and 169.5, C). The remaining three degrees of unsaturation again identified **3** as a tricyclic diterpenoid. The molecular framework was established by ^1^H-^1^H COSY and HMBC correlations (Figure 1). Comparison of the NMR data of **3** with those of the known compound sclerophytin E [27] revealed that **3** is the C-12 hydroxylated derivative of sclerophytin E. The stereochemistry of compound **3** was determined by the NOESY spectrum as shown in Figure 2.

The HRESIMS (*m*/*z* 461.2518 [M + Na]^+^) of krempfielin M (**4**) established the molecular formula of C_24_H_38_O_7_. The ^1^H and ^13^C NMR spectral data of **4** (Table 1) revealed that the structure of metabolite **4** should be similar to that of **3**, as the ^1^H NMR spectral data of **4** are almost identical with those of **3** except for the presence of one additional acetyl group (δ_H_ 2.06, s, 3H) in **4**. Furthermore, the placement of an acetoxy group at C-12 was established by the HMBC experiment which showed correlations from an oxymethine (δ_H_ 5.44, H-12) and acetate methyl (δ_H_ 2.06) to the ester carbonyl carbon appearing at δ_C_ 170.3 (C) (Figure 1). The NOE correlations of **4** also showed that the stereochemistry of this metabolite is identical with that of **3**. Thus the structure of metabolite **4** was determined.

The *in vitro* anti-inflammatory effects of the diterpenoids **1**–**6** were tested by examining the inhibitory activity of these compounds toward the release of elastase in *N*-formyl-methionyl-leucyl-phenylalanine/cytochalasin B (FMLP/CB)-induced human neutrophils cells (Table 2). At a concentration of 10 μM, all of these compounds could not significantly reduce the expression of superoxide anion, relative to the control cells stimulated with FMLP/CB only. At the same concentration, compounds **2** and **4** were found to effectively inhibit the elastase release (45.51 ± 2.69% and 27.30 ± 5.42% inhibition, respectively) in the same FMLP/CB-stimulated cells.

**Table 2:** Effect of pure compounds on elastase release in FMLP/CB-induced human neutrophils.

Compound	Elastase		
	Inh%		IC_50_ (μM)
**1**	19.42 ± 7.89	*	>10
**2**	45.51 ± 2.69	***	>10
**3**	18.67 ± 5.75	*	>10
**4**	27.30 ± 5.42	**	>10
**5**	−0.32 ± 0.81		>10
**6**	6.15 ± 3.42		>10

Percentage of inhibition (Inh%) was measured at 10 μM. Results are presented as mean ± S.E.M. (*n* = 3 or 4); * *p* < 0.05, ** *p* < 0.01 and *** *p* < 0.001 compared with the control value.

## 3. Experimental Section

### 3.1. General Experimental Procedures

Melting point was determined using a Fisher-Johns melting point apparatus. Optical rotations were measured on a JASCO P-1020 polarimeter. IR spectra were recorded on a JASCO FT/IR-4100 infrared spectrophotometer. ESIMS were obtained with a Bruker APEX II mass spectrometer. The NMR spectra were recorded either on a Varian UNITY INOVA-500 FT-NMR and a Varian 400MR FT-NMR. Silica gel (Merck, 230–400 mesh) was used for column chromatography. Precoated silica gel plates (Merck, Kieselgel 60 F-254, 0.2 mm) were used for analytical thin layer chromatography (TLC). High performance liquid chromatography was performed on a Hitachi L-7100 HPLC apparatus with an octadecylsilane (ODS) column (250 × 21.2 mm, 5 μm).

### 3.2. Animal Material

*C. krempfi* was collected by hand using scuba off the coast of Penghu islands of Taiwan in June 2008, at a depth of 5–10 m, and stored in a freezer until extraction. A voucher sample (specimen No. 200806CK) was deposited at the Department of Marine Biotechnology and Resources, National Sun Yat-sen University*.*

### 3.3. Extraction and Separation

The octocoral (1.1 kg fresh wt) was collected and freeze-dried. The freeze-dried material was minced and extracted exhaustively with EtOH (3 × 10 L). The EtOH extract of the frozen organism was partitioned between CH_2_Cl_2_ and H_2_O. The CH_2_Cl_2_-soluble portion (14.4 g) was subjected to column chromatography on silica gel and eluted with EtOAc in *n*-hexane (0%–100% of EtOAc, stepwise) and then further with MeOH in EtOAc with increasing polarity to yield 41 fractions. Fraction 28, eluted with *n*-hexane–EtOAc (1:2), was rechromatoraphed over a reversed-phase column (RP-18) using acetone–H_2_O (10:1) as the mobile phase to afford six subfractions (A1–A6). Subfraction A3 was repeatedly separated by reverse phase HPLC (CH_3_CN–H_2_O, 1.4:1.1) to afford compound **1** (2.6 mg). Fraction 31, eluted with *n*-hexane–EtOAc (1:10), was rechromatoraphed over a silica gel open column using *n*-hexane–acetone (3:1) as the mobile phase to afford eight subfractions (B1–B8). Subfraction B5 separated by reverse phase HPLC (CH_3_CN–H_2_O, 1:1 to 1:1.6) to afford compounds **2** (5.1 mg), **3** (2.8 mg), **5** (52.3 mg) and **6** (13.4 mg). Subfraction B6 by reverse phase HPLC (CH_3_CN–H_2_O, 1:1.5) to afford compounds **4** (8.4 mg). 

#### 3.3.1. Krempfielin J (**1**)

Colorless oil; [α]^23^_D_ = +52 (*c* 0.85, CHCl_3_); IR (neat) ν_max_ 3479, 2958, 2930, 2872, 1737, 1644, 1463, 1369, 1244, and 1100 cm^−1^; ^13^C and ^1^H NMR data, see Table 1; ESIMS *m*/*z* 417 [M + Na]^+^; HRESIMS *m*/*z* 417.2616 [M + Na]^+^ (calcd. for C_23_H_38_O_5_Na, 417.2617).

#### 3.3.2. Krempfielin K (**2**)

White powder; mp 162–163 °C; [α]^25^_D_ = −58 (*c* 1.5, CHCl_3_); IR (neat) ν_max_ 3444, 2963, 1731, 1651, 1464, 1380, 1240, 1177, 1087, and 1045 cm^−1^; ^13^C and ^1^H NMR data, see Table 1; ESIMS *m*/*z* 505 [M + Na]^+^; HRESIMS *m*/*z* 505.2780 [M + Na]^+^ (calcd. for C_26_H_42_O_8_Na, 505.2777).

#### 3.3.3. Krempfielin L (**3**)

Colorless oil; [α]^25^_D_ = +26 (*c* 0.8, CHCl_3_); IR (neat) ν_max_ 3419, 2958, 1734, 1457, 1369, 1246, 1063 and 1026 cm^−1^; ^13^C and ^1^H NMR data, see Table 1; ESIMS *m*/*z* 419 [M + Na]^+^; HRESIMS *m*/*z* 419.2407 [M + Na]^+^ (calcd. for C_22_H_36_O_6_Na, 419.2409).

#### 3.3.4. Krempfielin M (**4**)

Colorless oil; [α]^25^_D_ = +24 (*c* 2.4, CHCl_3_); IR (neat) ν_max_ 3452, 2960, 1736, 1435, 1370, 1243, 1199, 1075, and 1024 cm^−1^; ^13^C and ^1^H NMR data, see Table 1; ESIMS *m*/*z* 461 [M + Na]^+^; HRESIMS *m*/*z* 461.2518 [M + Na]^+^ (calcd. for C_24_H_38_O_7_Na, 461.2515).

### 3.4. *In Vitro* Anti-Inflammatory Assay—Superoxide Anion Generation and Elastase Release by Human Neutrophils

Human neutrophils were obtained by means of dextran sedimentation and Ficoll centrifugation. Measurements of superoxide anion generation and elastase release were carried out according to previously described procedures [28,29]. LY294002, a phosphatidylinositol-3-kinase inhibitor, was used as a positive control for inhibition of superoxide anion generation and elastase release with IC_50_ values of 1.88 ± 0.45 and 4.12 ± 0.92 μM, respectively. Briefly, superoxide anion production was assayed by monitoring the superoxide dismutase-inhibitable reduction of ferricytochrome c. Elastase release experiments were performed using MeO-Suc-Ala-Ala-Pro-Val-*p*-nitroanilide as the elastase substrate [30].

## 4. Conclusions

New eunicellin-based diterpenoids were isolated together with known ones from the soft coral *Cladiella krempfi.* Compounds **2** and **4** could significantly inhibit the release of elastase in FMLP/CB-induced human neutrophils. Thus, compounds **2** and **4**, in particular **2**, could be promising anti-inflammatory agents and may warrant further biomedical investigation.
